# Supplementary material for: Identification of Targetable Lesions in Anaplastic Thyroid Cancer by Genome Profiling
Source: Cancers (Basel). 2019 Mar 22;11(3):402. doi: 10.3390/cancers11030402 (PMC6468430; doi:10.3390/cancers11030402)
Supplement: Supplementary file 1 [file cancers-11-00402-s001.zip › cancers-454918-suppl-final/cancers-454918-suppl-final.pdf]

Supplementary Materials: Identification of Targetable Lesions in Anaplastic Thyroid Cancer by Genome Profiling

Naveen Ravi, Minjun Yang, Sigurdur Gretarsson, Caroline Jansson, Nektaria Mylona, Saskia R. Sydow, Eleanor L. Woodward, Lars Ekblad, Johan Wennerberg and Kajsa Paulsson

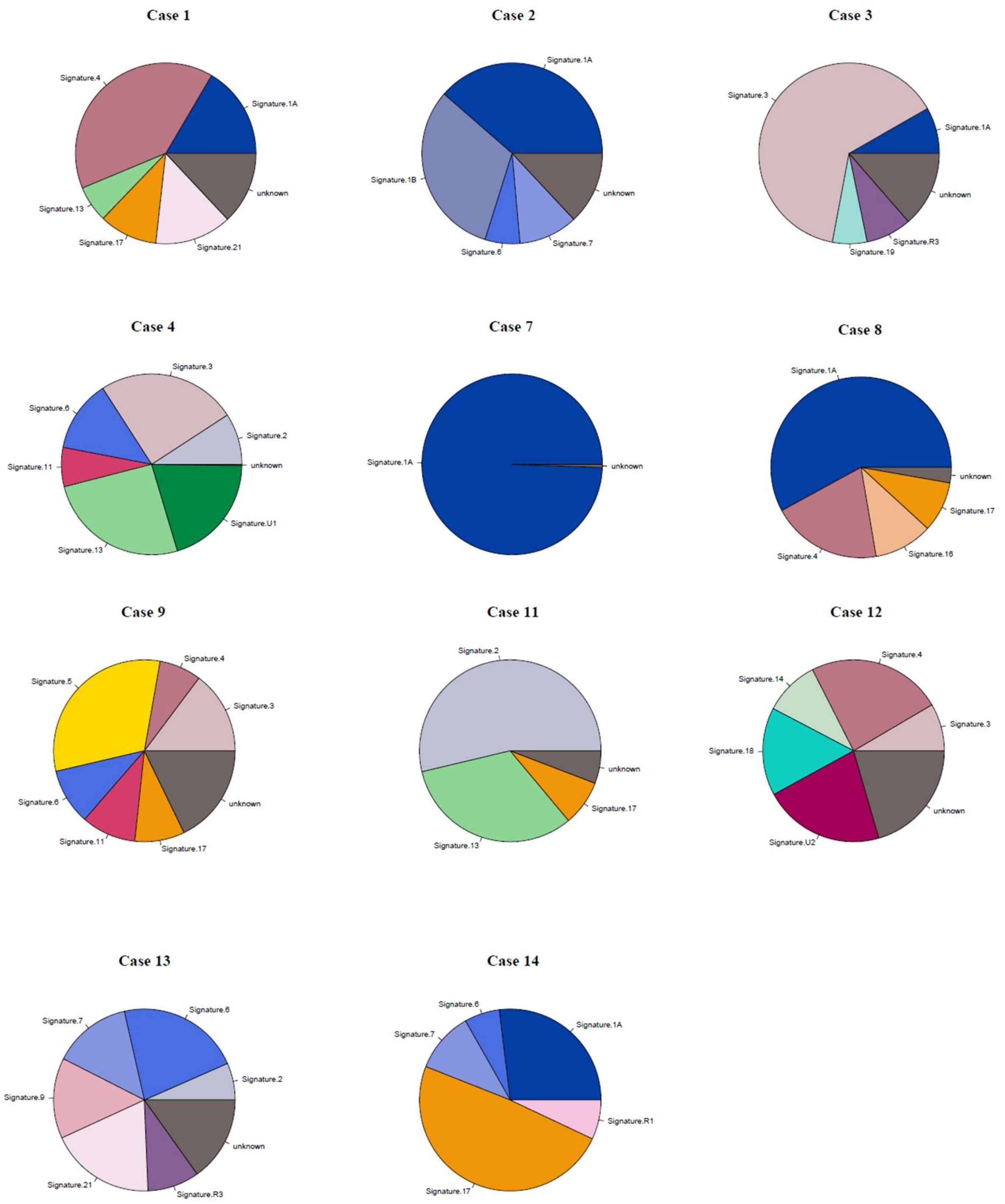

Figure S1. Mutation signatures of 11 primary anaplastic thyroid cancer cases, showing heterogeneity in the involved mutational processes.

© 2019 by the authors. Licensee MDPI, Basel, Switzerland. This article is an open access article distributed under the terms and conditions of the Creative Commons Attribution (CC BY) license (http://creativecommons.org/licenses/by/4.0/).
